# Forgotten Little Words: How Backchannels and Particles May Facilitate Speech Planning in Conversation?

**DOI:** 10.3389/fpsyg.2020.593671

**Published:** 2020-11-06

**Authors:** Birgit Knudsen, Ava Creemers, Antje S. Meyer

**Affiliations:** ^1^Max Planck Institute for Psycholinguistics, Nijmegen, Netherlands; ^2^Donders Institute for Brain, Cognition and Behaviour, Radboud University, Nijmegen, Netherlands

**Keywords:** conversation, turn taking, backchannel, speech planning, gap duration

## Abstract

In everyday conversation, turns often follow each other immediately or overlap in time. It has been proposed that speakers achieve this tight temporal coordination between their turns by engaging in linguistic dual-tasking, i.e., by beginning to plan their utterance during the preceding turn. This raises the question of how speakers manage to co-ordinate speech planning and listening with each other. Experimental work addressing this issue has mostly concerned the capacity demands and interference arising when speakers retrieve some content words while listening to others. However, many contributions to conversations are not content words, but backchannels, such as “hm”. Backchannels do not provide much conceptual content and are therefore easy to plan and respond to. To estimate how much they might facilitate speech planning in conversation, we determined their frequency in a Dutch and a German corpus of conversational speech. We found that 19% of the contributions in the Dutch corpus, and 16% of contributions in the German corpus were backchannels. In addition, many turns began with fillers or particles, most often translation equivalents of “yes” or “no,” which are likewise easy to plan. We proposed that to generate comprehensive models of using language in conversation psycholinguists should study not only the generation and processing of content words, as is commonly done, but also consider backchannels, fillers, and particles.

## Introduction

Everyday conversation consists of turns delivered by two or more speakers. Turns are not pre-planned and vary greatly in content and duration; yet, they are well coordinated in time (e.g., Sacks et al., 1974). Levinson (2016) reviewed evidence from several corpora (Stivers et al., 2009; Heldner and Edlund, 2010; Levinson and Torreira, 2015) and characterized the turn-taking system in the following way: “The system is highly efficient: less than 5% of the speech stream involves two or more simultaneous speakers (the modal overlap is less than 100 ms long), the modal gap between turns is only around 200 ms, and it works with equal efficiency without visual contact” (p. 6). The aim of the present article is to contribute to our understanding of the remarkable efficiency of the turn-taking system. We make a methodological point: To understand how conversation works, psycholinguists need to consider both the timing of turns and their content. Specifically, they need to study not only the production and comprehension of nouns and verbs, but also that of “little words,” especially backchannels. This may be an obvious point, but it has, in our opinion, not received sufficient attention in the field.

To return to turn taking in conversation, the short gaps in conversation stand in marked contrast to the much longer verbal response latencies observed in psycholinguistic experiments. For instance, participants typically need at least 600 ms to name common objects (e.g., Indefrey and Levelt, 2004), and they often need more than a second to begin to describe a scene in an utterance such as “the dog is chasing the mailman” (e.g., Griffin and Bock, 2000; Konopka, 2012). How can interlocutors in conversation respond to each other so fast? An obvious answer is that they do not await the end of their partner’s turn, but begin to plan their utterance earlier and then launch it at the appropriate time. This proposal is at the heart of the much cited model of conversational turn taking proposed by Levinson and Torreira (2015) see also Levinson (2016). In this model, interlocutors aim to grasp the speech act and gist of the partner’s utterance as early as possible and immediately begin to plan a response. While doing so, they listen to the interlocutor and aim to predict the end of their turn. When it is imminent, they launch the planned response. To illustrate, if your friend describes preparations for her birthday party and says “‘It’s on Sunday, would you like to…,” you can anticipate an upcoming invitation, prepare a response, and launch it well before the end of the utterance (“…come?”).

The Levinson and Torreira model explains the short gap durations by postulating linguistic dual-tasking, i.e., speech planning during listening. This should be a hard task because both listening and speech planning require processing capacity (e.g., Ferreira and Pashler, 2002; Strayer et al., 2003; Kubose et al., 2006; Cook and Meyer, 2008; Becic et al., 2010; Cleland et al., 2012; Boiteau et al., 2014), and because concurrent speech planning and listening may interfere with each other due to the activation of shared or tightly linked linguistic representations (e.g., Schriefers et al., 1990; Paucke et al., 2015; Fargier and Laganaro, 2016; Fairs et al., 2018). Thus, in conversation speakers must find suitable ways of dividing their processing resources across these processes and deal with interference between planned and heard words (e.g., Barthel and Sauppe, 2019, for further discussion). Much of the relevant empirical work has focused on the question whether speakers indeed begin to plan their utterances while listening to another person. The results strongly support this assumption. To illustrate, in a seminal study, Bögels et al. (2015) asked participants to respond as quickly as possible to general knowledge questions. They found that the participants responded faster when the cue to the answer appeared early in the question, as in “Which character, also called 007, appeared in the famous movie?” than when it appeared later, as in “Which character from the famous movies is also called 007?” (see also Bögels et al., 2018). Similar results were obtained in a study by Corps et al. (2018), where participants answered knowledge questions and questions about their personal experience [e.g., “Have you ever been to the city of Paris?” (late cue) versus “Are dogs your favorite animals?” (early cue); see also Corps et al., 2019], and in several studies where participants and confederates took turns describing objects on their screens (e.g., Sjerps and Meyer, 2015; Barthel et al., 2016; Meyer et al., 2018; Sjerps et al., 2020). All of these studies found faster responses when the cue to the answer appeared early rather than late in the question, indicating that, when possible, participants began to plan the answer during the question.

While all of these studies showed that response planning during listening is possible, they also clearly demonstrated that this alone could not be the key to smooth conversing. This is because in most studies, the participants’ average response latencies were considerably longer than the gap durations in corpora of conversational speech. For instance, in the early cue condition of the study by Bögels et al. (2015), the average response latency was 640 ms, in the study by Corps et al. (2018, Experiment 2b) it was 484 ms, and in the study by Barthel et al. (2016) it was 806 ms. Latencies almost as short as those reported for conversation (320 ms) were only seen in the study by Meyer et al. (2018), when participants said “yes” or “no” in response to questions about objects on their screens (“Do you have a green sweater?”). Given other laboratory studies on the time required to plan words and sentences (e.g., Indefrey and Levelt, 2004; Konopka, 2012), these latencies are not surprising, but the discrepancy to gap durations in conversation indicates that there must be factors that facilitate fast responding in conversation but are absent in laboratory settings. Many potentially important factors come to mind. For instance, in everyday conversation, interlocutors can build upon common ground, i.e. shared knowledge about the concepts discussed, which facilitates mutual comprehension and speech planning (e.g., Clark, 1996; Brown-Schmidt et al., 2015). Relatedly, as highlighted in the framework for conversation proposed by Pickering and Garrod (2004) and Garrod and Pickering (2009), interlocutors can align with each other, i.e., prime each other at multiple levels of representation (e.g., Segaert et al., 2016; Branigan and Pickering, 2017). Finally, participants in face-to-face conversation can draw on multi-modal, i.e., visual as well as auditory, information, whereas in the lab often only the auditory channel is available (Holler and Levinson, 2019).

The psycholinguistic work on conversation just mentioned has largely focused on the way speakers express conceptual content, i.e., pre-verbal messages, in spoken words or sentences and on the way addressees understand these utterances. Consistent with the bulk of experimental psycholinguistic work in other areas, the studies mainly concerned the production and processing of content words, usually nouns, phrases, or short sentences. Complementing this work, the present article concerns a feature of conversational speech that sets it apart from “lab speech,” the utterances elicited in typical laboratory settings: the presence of backchannels (BCs hereafter). We propose that BCs can be produced and responded to without much linguistic dual-tasking and may thereby contribute substantially to smooth conversing. We explain this in the following sections of this introduction. As we will also explain, utterance-initial fillers and some particles may have a similar function. In order to provide an estimate of how much speakers might gain from using BCs and particles, we analyzed two corpora, one in Dutch, and one in German, and determined their frequencies. We report the results in the empirical part of this article.

Backchannels are utterances such as English “mhm,” “uh-huh,” “wow,” “yeah,” and “really,” displaying comprehension of the speaker’s utterance and indicating that the addressee does not wish to take a full turn (e.g., Schegloff, 1982, 2000; Tolins and Fox Tree, 2016). They have also been called acknowledgment tokens (Jefferson, 1984), response tokens (Gardner, 2001), accompaniment signals (Kendon, 1967), or active listening responses (Simon, 2018). We call them BCs, following, among others, Tolins and Fox Tree (2014, 2016). We call the person providing them the addressee and their partner the speaker. We use the term “contribution” to refer to both regular turns and BCs.

Numerous studies have analyzed the forms, functions, and distributions of backchannels in conversation (e.g., Brunner, 1979; Clancy et al., 1996; Clark, 1996; Ward and Tsukahara, 2000; Bangerter and Clark, 2003; Clark and Krych, 2004; Norrick, 2012). For the present purposes, it is useful to distinguish between generic and specific backchannels (Goodwin, 1986; Bavelas et al., 2000; Tolins and Fox Tree, 2014, 2016). Generic backchannels are utterances such as “uh huh” or “yeah,” which display understanding and attention. They are sometimes called continuers (Schegloff, 1982; Goodwin, 1986; Stivers, 2008), as they invite the speaker to continue talking. Specific backchannels are utterances such as “woh” and “really.” They are sometimes called assessments (Goodwin, 1986; Bavelas et al., 2000), as they express the addressee’s response to the content of the utterance. Tolins and Fox Tree (2014) showed that readers of transcripts of dialogues asked provide the next turn responded differently to generic and specific backchannels: Generic backchannels encouraged the production of discourse-new information, whereas specific backchannels invited elaboration of earlier information.

As the BCs in a language differ in form and meaning, addressees must select the BC appropriate for the message they wish to convey. In this respect, planning BCs does not differ from planning other utterances. However, the set of common BCs in a language is small, they are used frequently, and their phonological forms tend to be simple. All of this should facilitate their selection and phonological planning compared to those of most content words (e.g., Jescheniak and Levelt, 1994; Meyer et al., 2003; Damian et al., 2010; Lau et al., 2019). Most importantly, the addressee producing a BC responds to the message provided by the speaker, rather than generating and encoding new conceptual content. Planning them during another utterance involves little linguistic dual-tasking because no new message is generated, and selecting and encoding a BC is, compared to the production of other utterances, less challenging.

Speakers usually respond to specific BCs by clarifying or augmenting their preceding utterance and to generic BCs by continuing their narrative. They must comprehend the BC, but crucially, the content of their next utterance does not depend on any conceptual content provided by the addressee. Instead, they continue to unfold their broad utterance plan or perhaps revise it. This means that the task set for participants in typical laboratory experiments, where they have to respond rapidly with content words or phrases to specific questions, may be different from the task in a natural conversation featuring BCs. This in turn implies that conclusions from lab experiments on the production or comprehension of content words and full sentences may not necessarily apply to conversations featuring much backchanneling.

A first step toward estimating how much BCs might alleviate the load from linguistic dual-tasking in conversation is to determine how often they occur. The aim of the corpus analyses reported below was to contribute to answering this question. We carried out these analyses because we found little information about the frequency of BCs in conversation in the literature. We briefly review the few relevant studies below.

Three studies reported the frequencies of BCs in task-oriented conversations. Bangerter and Clark (2003) reported between 78 and 101 BCs per 1000 words for corpora of conversation of participants jointly solving puzzle or construction tasks. The authors did not report the number of turns in the conversations. Knutsen et al. (2018b) reported 6397 BCs in a corpus of 83,173 words. This implies a rate of 78 BCs per 1000 words, which is similar to the lowest rate reported by Bangerter and Clark (2003). The proportion of turns including a BC was 61.8%. In a related study, where one participant (the Matcher) placed objects in a grid according to instructions given by another participant (the Director), Knutsen et al. (2018a) found that most placements of objects, which could include several turns, featured BCs by both the Director (in 61% of the cases) and the Matchers (in 92% of the placements). Thus, BCs occur frequently in task-oriented conversation. However, the studies do not report which proportions of the contributions consisted exclusively of BCs. Such contributions are most relevant to the current argument, as they do not include any new conceptual content to be formulated and responded to.

We found three studies reporting rates of BCs in conversations where participants just talked to each other without a specific task. White (1989) reported that in conversations held in American English, a generic BC appeared every 37 words; the rate of generic BCs was more than twice as high in conversation held in Japanese (see also Kita and Ide, 2007). The author does not report which proportion of the contributions consisted only of a BC, without additional words. Turkstra et al. (2003) reported that about 20% of the contributions in a corpus of conversations among adolescents included a generic BC, but could also include other materials. Finally, Jurafsky et al. (1997) reported that 19% of the contributions in the Switchboard corpus of English conversations were generic BCs, such as “uh-uh,” and 1% were specific BC, such as “okay.” This is the only study we found that provided the information directly relevant to our argument, i.e., the proportion of contributions that consisted only of a BC and no other materials.

To obtain new empirical evidence about the rates of BCs in conversation, we analyzed two corpora of conversational speech, one German, one Dutch. Our aim was to determine which proportions of the contributions consisted exclusively of a BC. These are the utterances discussed above, where the cognitive load due to linguistic dual-tasking should be low.

During the corpus analyses we noted that many contributions began with fillers such as “hm,” sometimes called filled pauses. These utterances are phonetically similar to BCs and often transcribed in the same way, but they are functionally distinct from BCs, as they do not encourage another speaker to continue talking, but constitute the onset of the current speaker’s contribution. However, they share with BCs that they are relatively easy to plan, since they are short and frequent and do not introduce new conceptual content. They result from a delay in the continuation of the utterance (e.g., Fox Tree and Clark, 1997; Clark and Wasow, 1998; Fox Tree, 2001; Clark and Fox Tree, 2002; Fraundorf and Watson, 2014; but see O’Connell and Kowal, 2005) and may alert the listener to upcoming processing difficulty (e.g., Arnold et al., 2003; Bosker et al., 2014; but see Corley and Hartsuiker, 2011). Fillers also share with BCs that they may alleviate the cognitive load from linguistic dual-tasking. The filler itself may be planned during the preceding contribution, but its articulation and any following pause buy the speaker some time for utterance planning, after the offset of the partner’s contribution. We also noted that many contributions began with closed class items, most often with translation equivalents of “yes” or “no.” These positive/negative particles have different discourse functions from fillers, but, like fillers, separate listening to another speaker’s contribution from planning utterance content. Thus, we report the proportions of utterances beginning with fillers and positive/negative particles and briefly discuss them below. All other contributions are referred to as “remaining contributions” below.

Though we were primarily interested in the proportions of the different types of contributions just described, we also report the durations of the gaps between contributions. To allow for comparison to earlier work, we report the distribution of gaps for the entire corpora. In addition, we report the average and median gap durations for each of the types of contributions just described. We stress that we had no specific hypotheses about the corresponding gap durations. Our proposal is that “little words,” BCs, fillers, and some particles, are easy to plan and respond to and reduce the need for linguistic dual-tasking, but this does not necessarily translate into fast utterance onsets. One might speculate that BCs should occur earlier than other contributions as they are easy to plan. However, a study by Roberts et al. (2015) did not find support for this hypothesis. Most likely, this is because speakers do not produce BCs as fast as they can but when such feedback to the speaker is deemed appropriate. As Roberts et al. (2015) have shown, gap durations in conversations are determined by many linguistic and non-linguistic variables, each with a small effect. Therefore, we did not expect to find large differences between the gap durations for the contributions we examined in our relatively small corpora. To reiterate, gap durations were not our main interest, but given that they resulted almost “for free” from our analyses, they are included in the tables and briefly discussed. Below, we first describe the two corpora, the analyses and results, and then offer a discussion of all relevant findings.

## Study 1: The Dutch IFADV Corpus

### Materials

The Dutch corpus IFADV (Instituut voor Fonetiek Amsterdam Dialogue Video) was prepared and made publicly available by van Son et al. (2008; accessible via https://www.fon.hum.uva.nl/IFA-SpokenLanguageCorpora/IFADVcorpus/). The corpus includes 20 recorded and annotated 15-min conversations of pairs of well-acquainted Dutch speakers, aged between 12 and 72 years, characterized as “good friends, relatives or long-time colleagues.” Three speakers participated twice with different partners. The participants talked about topics of their choice in a studio environment with audio and video recording equipment in view. The conversations were transcribed manually. Praat software (Boersma, 2001) was used for speech analysis. We used the transcripts of the conversations, rather than the audio-files, along with the time stamps for the onsets and offsets of contributions. Speaker changes were traceable in the corpus by reference to participant codes. The coding of the contributions was carried out by the first author and checked by the third author. Both are highly proficient bilingual speakers of German (first language) and Dutch.

### Analyses and Results

We parsed the corpus into contributions, defined by speaker changes, and computed the gap durations between them by subtracting the end of the contribution of one speaker from the onset of the contribution of the following speaker. Positive gap durations indicate that contributions followed each other and negative gap durations indicate that they overlapped.

We excluded all contributions containing noise, such as laughter and cough (transcribed as “ggg”), words not understood by the transcriber (transcribed as “xxx” and “^∗^x”) as well as slips of the tongue (transcribed as “^∗^u”) and interrupted words (transcribed as “^∗^a”). We excluded these contributions because their functions in the conversations could often not be established. Together the exclusions accounted for 20% of the contributions. As the categories “ggg,” “xxx,” and “^∗^a” were not mutually exclusive, the total rate of excluded utterances is less than the sum of the three rates.

The following analyses were based on the remaining 5645 contributions. To identify BCs, we first selected three types of contributions: (1) contributions consisting only of generic BCs, such as “hum” or “oh,” (2) contributions consisting of single words, and (3) contributions consisting of combinations of the two types of utterances (e.g., “ja, uhum”). Not all of these contributions were BCs. To exclude utterances that were not BCs, we considered the context and discarded contributions that were, for instance, answers to questions or completions of the other speaker’s contribution. In addition, we considered the word meanings. BCs should be relatively bland in meaning, as is the case for “really” or “cool.” When the two coders did not agree, a contribution was not considered a BC. A full listing of the types of contributions that we categorized as BCs, along with their frequencies, appears in the Supplementary Appendix. As Table 1 shows, together the three types of BCs accounted for 19% of the contributions with single words being most frequent.

**TABLE 1:** Frequencies (*n*) and proportions (%) of different types of contributions in the IFADV corpus with associated mean and median gap durations in seconds, 95% confidence interval of the means.

Contribution	*n*	%	Mean	95% CI		Median
**Excluded contributions**						
(1) Noise, laughter, not understood	945	13	−0.212	−0.272	−0.151	−0.181
(2) Slips and interruptions	640	9	0.018	−0.053	0.088	0.093
Total	1447	20				
**Backchannels**						
(3) Generic BCs	127	2	−0.051	−0.228	0.126	0.103
(4) Single words	877	12	−0.178	−0.229	−0.126	−0.004
(5) Combinations	323	5	−0.316	−0.410	−0.222	−0.252
Total	1327	19	−0.184	−0.228	−0.139	−0.036

(6) Continuations-after-BC	1080	15	0.543	0.503	0.583	0.390
(7) Utterances beginning with filler	218	3	−0.032	−0.155	0.091	0.114
(8) Utterances beginning with particle	1080	15	−0.126	−0.174	−0.077	0.000
(9) Remaining contributions	1940	27	−0.081	−0.118	−0.043	−0.065
Grand Total	7092					

Next, we examined the contributions following BCs. The aim was to determine whether these contributions were indeed continuations of the previous speaker’s utterances. Two hundred forty seven of the relevant contributions were excluded from the analysis for the reasons given above or because the conversation ended with the preceding BC, or because contributions following a BC were BCs themselves. All other cases (81% of all utterances following a BC, corresponding to 15% of all contributions) were clearly identifiable by their grammatical structure and content as continuations of the earlier utterance by the same speaker. The fact that most contributions following BCs were continuations of the speaker’s earlier turn validates our categorization of BCs as such. It also means that in total 34% of the contributions in the corpus (equivalent to 43% of the contributions included in the analyses) were utterances that could be produced with little linguistic dual-tasking because they were BCs or continuations of earlier utterances.

This left 3238 contributions, which were neither BCs nor utterances following them. Two hundred eighteen of them (3% of the contributions) began with a filler, such as “eh,” and 1080 of them (15%) began with forms of the positive/negative particles “ja” or “nee” (see Supplementary Appendix for a listing). These contributions are important here because fillers and particles allow speakers to begin to speak without planning much of the utterance content while listening to the interlocutor.

Table 2 provides summary information about the gaps in the two corpora analyzed here and, for comparison, the larger Dutch corpus (CGN, Corpus Gesproken Nederlands) by Heldner and Edlund (2010), which is often referred to in psycholinguistic studies of gap durations. As can be seen, the mean and median gap durations of the IFADV corpus are quite similar to those in the CGN.

**TABLE 2:** Descriptive statistics for gap durations (in milliseconds) in the IFADV, the German corpus (GECO), and the Dutch Corpus Gesproken Nederlands (CGN, Heldner and Edlund, 2010).

	IFADV	GECO	CGN
Mean	9	−91	8
SD	715	329	661
Median	71	−105	111
Skewness	−0.22	0.35	−0.5
Std. error skewness	0.03	0.02	0.01
Kurtosis	0.31	4.92	0.7
Std. error kurtosis	0.07	0.03	0.02
*n*	5436	21,676	43,374

*SD, standard deviation; n, number of observation; gap durations in milliseconds; Std. Error skewness (√6/N); Std. Error kurtosis (√24/N). The number of contributions and average gap durations in this table and in Tables 1 and 3 do not fully match because contributions in categories (1) and (2) of Tables 1 and 3 were not included here, and gaps with absolute durations exceeding 2 s were likewise excluded to achieve consistency with Heldner and Edlund’s treatment of the data.*

Table 1 shows the gap durations for each of the contribution type discussed above. The average gap durations were mostly negative and similar across contribution types. The only exception were the continuations after BCs, which were preceded by relatively long positive gaps. Inspection of the relevant sequences revealed that the addressee’s BC often began and ended during a speaker’s contribution, so that the gap measured from the offset of the BC to the speaker’s next contribution included the final part of the speaker’s contribution and any following pause. BCs were produced, on average, more than 100 ms earlier than the “remaining contributions,” i.e., the utterances that did not begin with a filler or yes/no particle [category (9) in the table]. The 95%-confidence intervals for the two types of contributions did not overlap, which indicates that the difference in gap durations was statistically significant. Based on results reported by Roberts et al. (2015) we had not expected to see such a difference, but it is consistent with the assumption that BCs are relatively fast to produce.

## Study 2: The German Corpus

### Materials

The German corpus (GECO, Schweitzer and Lewandowski, 2013) consists of 46 dialogues. In 22 of them, the participants could not see each other (unimodal condition), and in the remaining 24 dialogues they were facing each other (multimodal condition). The analyses presented below concern the latter dialogues. The participants were eight women, aged between 20 and 30 years, who did not know each other. Each of them talked to three different partners. To ease conversation a list of potential topics was provided, but participants were free to choose other topics as well. Each conversation lasted for approximately 25 min. The dialogues were transcribed and analyzed using Praat software (Boersma, 2001). In the transcripts prepared by the authors, speaker changes are traceable through specific participant numbers. Gap durations were calculated as described above by subtracting the offset time of a contribution from the onset time of the next contribution.

### Analyses and Results

Contributions were categorized in the same way as described above for the IFADV corpus. Sixteen percent of the contributions were excluded because they included noise or laughter, were not fully transcribed, or contained slips or interrupted words. BCs were identified in the same way as for the IFADV corpus, though the specific utterances classified as BCs differed in the two languages. A listing of the contributions categorized as BCs and fillers appears in the Supplementary Appendix. Sixteen percent of the contributions were BCs, which closely corresponds to the rate of 19% obtained for the IFADV corpus.

Ninety percent of the contributions following BCs (corresponding to 14% of all contributions in the corpus) were continuations of the utterance the speaker had begun before the BC. The remaining 10% of the contributions were excluded from the analyses for the reasons given above, or they were themselves BCs. Thus, as expected and observed for the IFADV corpus, the speakers continued their turn after the BC.

Inspection of the other contributions in the corpus revealed that 518 of them (2% of all contributions) began with a filler and 1463 (6% of all contributions) with a yes/no particle (see Supplementary Appendix for a listing of fillers and relevant particles). As explained earlier, in these contributions, the cognitive load due to linguistic dual-tasking may be reduced, relative to the planning of other utterances, because most of the conceptual content and linguistic form of the utterance is likely to be planned after the end of the other speaker’s contribution.

Table 2 provides statistical information about the gap durations in this corpus. With mean and median just below 0 ms, the gaps are somewhat shorter than those reported for the CGN. Table 3 shows that the mean and median gap durations were slightly negative for all contribution types apart from contributions beginning with fillers or particles, which were preceded by short positive gaps. As in the IFADF corpus, gaps before BCs were significantly shorter, by 59 ms in this case, than gaps before “remaining contributions.”

**TABLE 3:** Frequencies (*n*) and proportions (%) of different types of contributions in the GECO corpus with associated mean and median gap durations in seconds, 95% confidence interval of the means.

Contribution	*n*	%	Mean	95% CI		Median
**Excluded turns**						
(1) Noise, laughter, not understood	3757	14	−0.306	−0.335	−0.278	−0.130
(2) Slips, interruptions	572	2	0.004	−0.032	0.041	−0.043
Total	4266	16				
**Backchannels**						
(3) Generic BCs	1825	7	−0.291	−0.324	−0.258	−0.225
(4) Single word	1690	6	−0.145	−0.168	−0.122	−0.160
(5) Combinations	583	2	−0.092	−0.138	−0.045	−0.065
Total	4098	16	−0.202	−0.221	−0.184	−0.175

(6) Continuations-after-BC	3699	14	−0.023	−0.034	−0.013	−0.075
(7) Utterances beginning with filler	518	2	0.046	0.013	0.079	0.020
(8) Utterances beginning with particle	1463	6	0.062	0.041	0.082	0.010
(9) Remaining contributions	12,047	46	−0.143	−0.151	−0.134	−0.115
Grand total	26,091					

## Discussion

In a seminal paper, Levinson and Torreira (2015) proposed that swift turn taking in conversation resulted from linguistic dual-tasking: The interlocutors listen to each other and simultaneously prepare their own utterances such that by the end of one speaker’s turn, the next speaker is ready to speak. Given the average gap durations of 300 ms or less, which have been consistently reported across corpora (e.g., Stivers et al., 2009; Heldner and Edlund, 2010; Roberts et al., 2015), utterance preparation before the end of the preceding turn indeed appears to be necessary. However, from a psycholinguistic perspective this conclusion is puzzling because speech planning during listening should be effortful and inefficient. This is because speaking and listening both require processing capacity, and because they involve access to shared or closely linked representations, which may interfere with each other. Indeed, laboratory experiments where participants must combine listening with speech planning have shown that linguistic dual-tasking is effortful and that both speech planning and listening are less efficient in dual- than in single-task settings (e.g., Boiteau et al., 2014; Sjerps and Meyer, 2015). An important research issue therefore is why conversations appear to be so much more efficient and effortless than listening and speaking in the lab.

In this article we draw attention to the fact that natural conversation, but not the speech elicited in typical laboratory experiments, includes backchannels (BCs). This is important because BCs may reduce the need for linguistic dual-tasking. Speakers must select BCs, just like content words. This is because the BCs available in a language differ in meaning and form, and speakers have to choose the BC reflecting the meaning they wish to convey, for instance “hm” as a generic backchannel or “cool” as a more specific one. However, as the set of available BCs is small, and as they are short and frequent, they should be much easier to plan than content words. Most importantly, BCs do not introduce new conceptual content but reflect the addressee’s understanding of the speaker’s turn. The dual-task load arising when, for instance, a person thinks of an answer to a quiz question while still listening to the final part of the question, does not arise when a BC is planned.

In the present study we examined how often BCs occurred in two corpora of casual conversation, the Dutch IFADV corpus, consisting of conversations between speakers who knew each other well, and the German corpus (GECO), consisting of conversations between strangers. We found similar rates of BCs in the two corpora, 19% in the IFADV and 15% in the German corpus. These rates match the rate reported by Jurafsky et al. (1997) for the English Switchboard corpus (19%), which consists of recorded telephone conversations between strangers. All of these conversations were held specifically for recording and analysis, and it remains to be seen how often BCs occur in conversations in other contexts and different languages. However, as speakers were not instructed to talk in particular ways, we think that 15% is a reasonable estimate of the proportion of contributions to casual conversation in these Germanic languages that are BCs.

We determined not only the rates of BCs, but also examined the contributions following them. Most of them were continuations of utterances by the previous speaker. This is unsurprising because the primary function of BCs is to encourage the current speaker to continue talking and, complementary to this, to indicate that the addressee does not wish to initiate a turn (e.g., Schegloff, 1982). Earlier work has shown that BCs affect what speakers will say next (e.g., Bavelas et al., 2000; see also Tolins and Fox Tree, 2016). For instance, a specific BC may encourage a speaker to revise their speech plan and elaborate on an earlier statement. Thus, speakers must engage in some linguistic dual-tasking when they process BCs, as they need to understand the BC and possibly revise their speech plan. However, they do not have to process, or try to ignore, further incoming speech while preparing their utterance. In terms of the complexity of the cognitive processes and processing load, this is an important difference to the typical laboratory situation, where speakers have to generate utterances while listening to ongoing speech (e.g., Bögels et al., 2015; Sjerps and Meyer, 2015; Corps et al., 2018; Barthel and Sauppe, 2019).

Evidently, these considerations concerning the processing load arising in sequences featuring BCs are speculative, as we did not measure and compare the cognitive load arising during the generation and processing of BCs and other utterances. This can be done in future work. The main point of the present article is to highlight that many contributions to a conversation, around 15% on our estimate, are BCs, and about the same number of contributions are responses to them, and as such differ markedly from the utterances typically induced in the lab.

The occurrence of BCs is not the only feature that distinguishes conversational from laboratory speech. Another feature is the occurrence of fillers, which are phonetically similar to BCs and therefore caught our attention. They occurred at the beginning of 3% of the contributions in the Dutch corpus and of 2% of the contributions in the German corpus. In experimental research, participants are usually asked not to produce fillers because they jeopardize the measurement of the onset latency for the following content word. In conversation, fillers can serve different purposes; most commonly they signal a delay in the production of the next part of the utterance (e.g., Fox Tree and Clark, 1997; Clark and Wasow, 1998; Clark and Fox Tree, 2002; see also Arnold et al., 2003). In addition, they may reduce the load from linguistic dual-tasking. This is because the speaker only needs to plan the filler while listening to their partner, and can generate the remainder of the utterance during the articulation of the filler and any following pause.

Finally, in the Dutch corpus a substantial proportion, 15%, of the contributions began with a positive/negative particle. Using such particles, just like fillers, leads to a temporal separation of listening and speech planning and therefore reduces the cognitive load arising from simultaneous listening and speech planning. In the German corpus, the proportion of contributions beginning with particles was, at 6%, much lower. It is not clear why the conversations differed in this respect. It is possible that turn-initial particles are overall used more frequently in Dutch than in German casual conversation, or that the difference was related to the differences in speaking styles adopted in conversations between friends (the Dutch sample) and strangers (the German sample).

In addition to the rates of different types of contributions we reported the gaps preceding them. For most types of contributions, the gaps were negative, meaning that speakers began to talk slightly before the end of the preceding turn. Exceptions were the continuations after BCs in the Dutch corpus (IFADV) and the utterances beginning with fillers or yes/no particles in the German corpus (GEKO). In both corpora, BCs were produced significantly earlier than the “remaining contributions,” i.e., the contributions that were not BCs, continuations after BCs, or contributions beginning with fillers or yes/no particles. This result is consistent with our view that BCs are easy to plan and produce. Nonetheless, we had not expected to find this difference because earlier studies had not found that BCs were placed particularly early (e.g., Roberts et al., 2015; see also Beňuš et al., 2011, for a detailed analysis of the timing of BCs and filled pauses in a single conversation). Based on this work we had assumed that the short planning times for BCs might affect the gaps preceding them, but that this influence would probably be too subtle to manifest in the gap durations. Contrary to this expectation, we observed that BCs were produced earlier than the “remaining contributions”.

The early placement of BCs contributes to the short average gap durations across all contributions. However, it does not, by itself, offer a solution to the riddle why speakers are so much faster to respond to each other in conversation than one would predict on the basis of the response latencies observed in laboratory experiments on conversational turn taking. The riddle would be solved if BCs were placed very early and most other contributions very late. However, such a pattern was not observed. Though BCs were produced particularly earlier, most other types of contributions also began before the end of the preceding turn. This contrasts sharply with the positive gap durations observed in laboratory experiments on conversation reviewed in the introduction (e.g., Bögels et al., 2015; Corps et al., 2018).

To explore what allowed the participants in the conversations to respond so fast to each other, we examined the first words of the “remaining contributions” in the IFADV corpus, i.e., the utterances that were not BCs or responses to them and did not start with a filler or yes/no particle. A syntactic and semantic analysis of these contributions is beyond the scope of the present article, but two observations seem worthwhile reporting. First, 1245 (74%) of the 1680 relevant contributions began with a closed class item, such as a determiner, pronoun, or conjunction. To put this differently, only about a quarter of the utterances began with a content word. Second, there were 1293 contributions in the set that consisted of at least three words, and of these a third (408 contributions) began with a sequence of three closed class items, such as “en ik was” (“and I was”) or “het was zo” (“it was such that”). How quickly speakers can plan individual closed class items or sequences of such items is unknown and probably depends on the type of items and their functions in the grammatical structure of the utterance. Nevertheless, given their high frequency, at least some function words may be faster to plan than content words. This is important because in earlier work, gap durations in conversation have often been compared to speech production latencies for nouns, specifically picture names (e.g., Garrod and Pickering, 2015; Levinson and Torreira, 2015; Barthel et al., 2016; Bögels et al., 2018; Barthel and Sauppe, 2019; Sjerps et al., 2020), and relative to these latencies gaps in conversation are very short. This comparison may be misleading as it ignores the difference in word class and any related differences in the speech planning processes.

More generally, experimental work on conversation should study all of the different types of utterances that interlocutors produce (see also De Ruiter and Albert, 2017; Meyer et al., 2018). To do so, researchers need to know what these utterances are. By reporting the rates of backchannels in two corpora of conversational speech the present work contributes to this body of knowledge. We think that backchannels, fillers, and particles deserve increased attention in psycholinguistics because they may be produced and processed differently from open class items and perhaps contribute substantially to the easy flow of conversation. Whether this is true can be explored in future work.

## Data Availability Statement

Publicly available datasets were analyzed in this study. The data of the IFADV corpus can be found here: https://www.fon.hum.uva.nl/IFA-SpokenLanguageCorpora/IFADVcorpus. The data of the GECO corpus can be requested here: https://www.ims.uni-stuttgart.de/en/research/resources/corpora/ims-geco/.

## Ethics Statement

Ethical review and approval was not required for the study on human participants in accordance with the local legislation and institutional requirements. The participants of both corpora provided their written consent to participate in the studies.

## Author Contributions

AM took the lead in developing the theoretical argument, planning the analyses, and writing the manuscript. BK carried out all analyses and contributed to writing and editing the manuscript. AC contributed to the development of the theoretical argument and to writing and editing the manuscript. All authors contributed to the article and approved the submitted version.

## Conflict of Interest

The authors declare that the research was conducted in the absence of any commercial or financial relationships that could be construed as a potential conflict of interest.
