# Supplementary material for: Forgotten Little Words: How Backchannels and Particles May Facilitate Speech Planning in Conversation?
Source: Front Psychol. 2020 Nov 6;11:593671. doi: 10.3389/fpsyg.2020.593671 (PMC7677452; doi:10.3389/fpsyg.2020.593671)
Supplement: Supplementary file 1 [file Data_Sheet_1.docx]

**Appendix**

Table A1. Listing and frequencies (n) of backchannels in the IFADV corpus

| **Backchannels** | **n** |
| --- | --- |
| *generic BCs* |  |
| ah, aha | 4 |
| goh | 3 |
| hum | 54 |
| jee, joh | 2 |
| kch | 5 |
| mmm | 5 |
| o. oh, oh? | 46 |
| uh, uhm, uhu | 7 |
| wauw, woh | 4 |
| Total | 130 |
| *single words* |  |
| cool | 1 |
| echt? | 3 |
| ja, ja?, jeetje | 769 |
| kijk | 2 |
| nee, nee?, nou, nou? | 70 |
| oké | 51 |
| precies | 2 |
| toch? | 5 |
| wat? | 1 |
| zeker, zo | 4 |
| Total | 908 |
| *combinations* |  |
| ah zo, ah oké | 2 |
| goh ja? | 1 |
| hum hum, hum? hum, hum ja, hum nee | 77 |
| ja? ah, ja daarom, ja hé, ja hum, ja inderdaad, ja ja, ja ja?, ja joh, ja klopt, ja nee, ja nou, ja oké, ja precies, ja tuurlijk, ja uhum, ja wow | 164 |
| mm-hu, mmm ja | 4 |
| nee ja, nee nee, nee o, | 25 |
| o oké, och jee, oh bah, oh God, oh goed, oh goh, oh hum, oh ja, oh jee, oh joh,  oh oh, oh oké, o ja, o ja?, o jee, oké ja, o nee, o precies | 51 |
| uh uh, uhu oké | 2 |
| Total | 326 |
| Grand Total | 1364 |

Table A2. Listing and frequencies (n) of fillers and positive/negative particles in the IFADV corpus

| **Fillers and particles** | **n** |
| --- | --- |
| *fillers* |  |
| ach, ah | 15 |
| h\e', hoi, hum | 27 |
| jo, joh | 2 |
| kch | 3 |
| mm, mwah | 3 |
| o, och, oh, oh? | 117 |
| uh, uhm | 50 |
| woh | 1 |
| Total | 218 |
| *particles* |  |
| ja, ja?, jawel | 894 |
| nee | 186 |
| Total | 1080 |
| Grand Total | 1298 |

Table A3. Listing and frequencies (n) of backchannels and in the German corpus (GECO)

| **Backchannels** | **n** |
| --- | --- |
| *generic BCs* |  |
| Aaah, Ach, Ah, Äh, Aha, Au | 52 |
| Ha, Hä?, Hah, Häh?, He, Hm, Hm?, Hmm, Hmmh, Ho, Hoho, Höho | 55 |
| Mh, Mhh, Mhhh, Mhm, Mhmhh, Mm, M-m, Mmh, M-mh, Mmm | 1683 |
| Na, Ne, Ne? | 3 |
| O, Och, Oh, Öh, Oha, Oooh, Ooooh | 31 |
| Uh, Ui | 2 |
| Woah, Wow | 12 |
| Yeah | 1 |
| Total | 1839 |
| *single words* |  |
| Achso | 9 |
| Cool | 37 |
| Doch, Doch? | 3 |
| Eben, Echt? | 38 |
| Geil, Gell?, Genau, Gut | 48 |
| Ja, Ja? | 1161 |
| Klar, Krass | 44 |
| Mja | 2 |
| Nee, Nee?, Nein, Nicht? | 40 |
| Oder? Ok, Okay | 317 |
| Schon, Schon?, Schön, Stimmt | 15 |
| Toll | 2 |
| Was? Wirklich? | 7 |
| Total | 1723 |
| *combinations* |  |
| Aaah cool, Ah cool, Ach cool, Ah gut, Ah ha, Ach ja, Ah ja, Ahja ja, Ahja klar,  Ah mhm, Ahja, mhm, Ah oh, Ach oh, Ah ok, Aha ok, Ach ok, Ach so, Ah super, Ach was? | 113 |
| Genau ja, Genau mhm | 7 |
| Hmm gut, Ha ja, Hm ja, Hmm mhm, Haha mhm, Hm mhm, Hoho mhm, Hm schön | 12 |
| Ja absolut, Ja cool, Ja dann, Ja doch, Ja eben, Ja genau, Ja gut, Ja hah, Jaja ja, Ja ja, Ja klar, Ja komisch, Ja krass, Ja mega, Ja mh, Ja mhm, Jee mhm, Ja mm Ja naja, Ja natürlich, Ja ne?, Ja nee, Ja nein, Ja oh, Ja ok, Ja schon, Ja schön, Ja total, Ja trotzdem, Ja übel, Ja voll, Ja Wahnsinn, Ja wuah | 276 |
| Krass ja | 3 |
| Mhm ah, Mh ah, Mhm cool, Mh cool, Mhm genau, Mhh hmm, Mh ja, Mhm ja,  M-m ja, Mmm ja, Mhm klar, Mh mh, Mhm mh, Mhm nein, Mhm oh, Mh ok, Mhm ok, Mhm schon | 118 |
| Na also, Nein genau, Na ja, Nie ja, Nee mhm,  Na schade, Na toll | 12 |
| O Gott, O je, O Wahnsinn, Och nein, Och mhm, Oh cool, Oh ja, Oh krass, Oh blöd, Oh nein, Oh ok, Öh ok, Ok ach, Ok hm, Ok ja, Ok mh, Ok mhm, Ok mm, Ok ne, Ok nein, Ok ok | 51 |
| So ja, So mhm, Stimmt ja | 3 |
| Voll cool | 1 |
| Wow heftig, Wow ok | 2 |
| Total | 598 |
| Grand Total | 4160 |

Table A4. Listing and frequencies (n) of fillers and positive/negative particles in the German Corpus (GECO)

| **Fillers and particles** | **n** |
| --- | --- |
| *(7) filler* |  |
| Aah, Ach, Achso, Ah, Äh, Aha, Ahja, Ahm, Ähm | 247 |
| Boah | 4 |
| Ey | 1 |
| Ma, Mh, Mhh, Mhm, Mm, M-m, Mmm | 192 |
| Ha, Hä, Hach, Hah, Hehe, Hm, Hmm, Hm-m | 25 |
| O, Och, Oh, Öh | 42 |
| Pff, Psch | 4 |
| Wow | 3 |
| Total | 518 |
| *(8) particles* |  |
| Ja, Ja?, Jaja, Jein | 1283 |
| Nee, Nein, Nein?, Nö | 180 |
| Total | 1463 |
| Grand Total | 1981 |
